# Magnetic resonance imaging-based deep learning for predicting subtypes of glioma

**DOI:** 10.3389/fneur.2025.1518815

**Published:** 2025-01-29

**Authors:** Zhen Yang, Peng Zhang, Yi Ding, Liyi Deng, Tong Zhang, Yong Liu

**Affiliations:** ^1^Department of Neurosurgery, The Second People’s Hospital of Hefei, Hefei Hospital Affiliated to Anhui Medical University, Hefei, China; ^2^Department of Neurosurgery, The Affiliated Hospital of Xuzhou Medical University, Xuzhou, China

**Keywords:** deep learning, IDH, glioma, 1p/19q, MRI

## Abstract

**Purpose:**

To explore the value of deep learning based on magnetic resonance imaging (MRI) in the classification of glioma subtypes.

**Methods:**

This study retrospectively included 747 adult patients with surgically pathologically confirmed gliomas from a public database and 64 patients from our hospital. Patients were classified into IDH-wildtype (IDHwt) (490 cases), IDH-mutant/1p19q-noncodeleted (IDHmut-intact) (105 cases), and IDH-mutant/1p19q-codeleted (IDHmut-codel) (216 cases) based on their pathological findings, with the public database of patients were divided into training and validation sets, and patients from our hospital were used as an independent test set. The models were developed based on five categories of preoperative T1-weighted, T1-weighted gadolinium contrast-enhanced, T2-weighted and T2-weighted fluid-attenuated inversion recovery (T1w, T1c, T2w and FLAIR) magnetic resonance imaging (MRI) of four sequences and mixed imaging of the four sequences, respectively. The receiver operating characteristic curve (ROC), area under the curve (AUC) of the ROC were generated in the jupyter notebook tool using python language to evaluate the accuracy of the models in classification and comparing the predictive value of different MRI sequences.

**Results:**

IDHwt, IDHmut-intact and IDHmut-codel were the best classified in the model containing only FLAIR sequences, with test set AUCs of 0.790, 0.737 and 0.820, respectively; and the worst classified in the model containing only T1w sequences, with test set AUCs of 0.621, 0.537 and 0.760, respectively.

**Conclusion:**

We have developed a set of models that can effectively classify glioma subtypes and that work best when only the FLAIR sequence model is included.

## Introduction

Glioma is the most common primary brain tumor (1), and the classification and definition of glioma are changing as people’s understanding of glioma continues to grow. In 2016, the WHO first proposed the importance of IDH and 1p/19q co-deletion status in the diagnostic classification of glioma (2), and in 2021, the WHO further clarified the role of IDH and 1p/19q co-deletion status in the diagnostic classification of glioma in the new version of the classification criteria for central nervous system tumors (3). The current general treatment principle for glioma is to surgically remove the maximum extent of the tumor as far as is safe (4, 5), followed by individualized treatment regimens such as radiotherapy and chemotherapy (6–8). However, the prediction of survival, the progression of the disease, the extent of surgical resection, the development of individualized treatment plans and the assessment of efficacy are all closely related to the type of glioma (9–12). Pre-operative knowledge of the patient’s tumor type can help patients to start a more precise and personalized treatment at the beginning of the disease. Currently, the assessment of the type of glioma is based on surgical or post-biopsy histopathology. However, pre-operative biopsy may be limited by the unavailability of tumor tissue, the fact that some of the tissue obtained is not representative of the whole tumor, the heterogeneity of the tumor and so on. Post-operative histopathology to assess the grade and type of glioma is also not effective in preoperative diagnosis and treatment planning of glioma. Therefore, a new method is needed to predict the grade and classification of gliomas.

Magnetic resonance imaging (MRI) is an effective method for diagnosing gliomas. Experienced clinicians can make a preliminary determination of the type of tumor by the location of the tumor, the peri-tumoural oedema and features such as calcification and necrosis within the tumor (13, 14), however, using this information to make predictions has limitations due to the lack of standard, quantifiable parameters. At the same time, certain microscopic features in MRI cannot be identified by the naked eye. In recent years, with the rapid development of computer software and hardware, deep learning has also been making breakthroughs, and automatic feature extraction has brought great improvements in terms of efficiency and performance, as well as good results in the medical field. Deep learning can automatically extract features from MRI (15) to help clinicians make auxiliary decisions.

Classification tasks are an important research direction for computer vision in deep learning, and in the clinic, accurate and effective classification of diseases has been a big problem for clinicians. With the continuous development of deep learning, deep learning is increasingly used in studies about disease classification. Chen et al. (16) used deep learning to effectively classify gliomas with and without the occurrence of MGMT promoter methylation. The present study aims to investigate the value of MRI-based deep learning for predicting diffuse glioma in adults, as preoperative classification of glioma has been a clinical challenge and there are few studies to predict the staging of glioma based on deep learning.

## Materials and methods

### Ethics statement

This retrospective study was approved by the ethics committee of our hospital (*XYFY2022-KL476-01*). Written informed consent was waived due to the retrospective nature of the study. As dataset 1, 2, 3 and 4 are freely and publicly available on public databases for viewing, downloading and use for scientific purposes, institutional review board approval and written informed consent are not required for this study.

### Patient selection

There are five datasets included in this study, dataset 1 contains 86 patients from TCGA-LGG (17) in TCIA, dataset 2 contains 88 patients from TCGA-GBM (18) in TCIA, dataset 3 contains 382 patients from UCSF-PDGM (19) in TCIA, dataset 4 contains data from The Erasmus Glioma Database (20) of 191 patients, and dataset 5 contained 64 patients from our hospital from September 2019 to October 2022. All data met the following inclusion criteria: (1) adult patients (age > 18) with pathologically confirmed primary glioma; (2) clear grade of glioma and IDH with 1p/19q co-deletion status; (3) inclusion of preoperative T1-weighted, T1-weighted gadolinium enhancement, T2-weighted and T2-weighted fluid-attenuated inversion recovery (T1w, T1c, T2w and FLAIR) MRI; (4) clear imaging. Exclusion criteria included: (1) history of brain tumor surgery or biopsy; (2) unclear WHO grade, IDH status, or 1p/19q co-deletion status for gliomas; (3) absence of preoperative T1w, T1c, T2w, and FLAIR; (4) poor quality of MRI scans, specifically images with significant artifacts that could not be accurately delineated for regions of interest (ROI) after consensus between two physicians. Since some of the TCGA-LGG and TCGA-GBM data were used in Brain Tumor Segmentation 2020 (BraTS20) (21), in this study, only that part of the data used in BraTS20 was selected for TCGA-LGG and TCGA-GBM.

We classified all patients into 3 categories based on the IDH and 1p/19q co-deletion status of each patient: IDH-wildtype (IDHwt), IDH-mutant/1p19q-noncodeleted (IDHmut-intact), and IDH-mutant/1p19q-codeleted (IDHmut-codel).

### MRI parameters

The MRI of the five data sets in this experiment were taken by different MRI scanners and each patient contained four MRI sequences, T1w, T2w, FLAIR and T1c. The specific MR parameters are shown in the Appendix.

### Pre-processing of images

In order to eliminate differences between the different images, all images were pre-processed in a uniform manner. The processing was as follows: (1) correction for bias magnetic fields was performed using N4ITK; (2) rigid registration was performed using the General Registration module in 3D Slicer, with T1C as the reference image; (3) skull stripping of the images was performed using the Swiss Skull Stripper module in 3D Slicer; and (4) the intensity normalization was performed using the Z-score standardization method.

### ROI drawing and segmentation

After the preprocessing steps, ROIs were delineated on the FLAIR sequence using 3D Slicer (v5.2.1). The boundaries of the ROIs, including the tumor, tumor necrosis, intratumoral calcification and peritumoral edema, were determined through consensus between two physicians (a neurosurgeon with 7 years of clinical experience and a resident with 3 years of clinical experience). In cases of disagreement or uncertainty, a senior attending physician made the final decision. Subsequently, the resident independently manually delineated the ROIs on all tumor-containing images. After completing the delineation on the FLAIR images, the defined regions were transferred to T1w, T2w, and T1c images to verify the accuracy of the ROI segmentation. The images were then cropped to exclude regions outside the ROI, and external voxel values were set to zero. It is noteworthy that in this study, the tumor was not subdivided into distinct subregions or areas. Instead, the entire tumor region on each image was treated as a single ROI. Prior to the ROI delineation and segmentation process, a third member of the research team removed all labels and tumor type annotations from the images and renumbered them with numeric identifiers to ensure anonymization.

In this study, data sets 1 and 2 were taken from the BraTS20 dataset and all data were aligned and cranially stripped and some data were ROI plotted, while data sets 3 and 4 were also aligned and cranially stripped and all data were ROI plotted. Therefore, data that had been aligned and cranially stripped were not re-aligned and cranially stripped, and data that had been ROI plotted were only checked but not re-plotted.

After image segmentation, all images from the four pulse sequences underwent uniform preprocessing, which included manually retaining tumor-containing slices and resizing the images to a consistent resolution of 224 × 224 pixels. These processed images were then used as input for the Convolutional Neural Network (CNN).

### CNN

**Figure 1:**
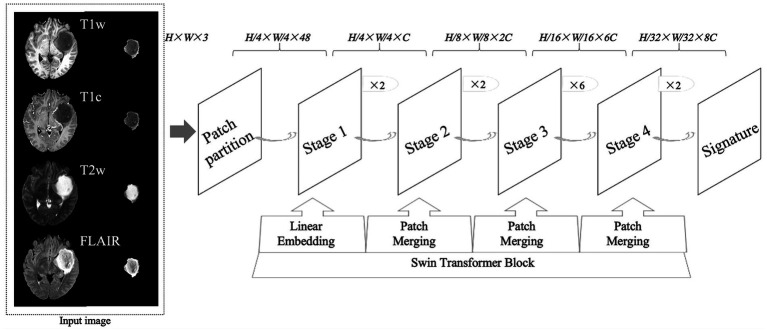
An overview of the research design, including the structure of convolutional neural networks. T1w, T1-weighted; T1c, T1-weighted gadolinium contrast-enhanced; T2w, t2-weighted; FLAIR, T2-weighted fluid-attenuated inversion recovery. The image size fed into the neural network was 224 × 224, where *H* represents the image height, *W* represents the image width, and 3 represents the image as a 3-channel RGB image.

The CNN model used for glioma classification in this study is the Swin Transformer, as shown in Figure 1. The training was conducted on the MMDetection platform, an open-source toolbox for object detection built on PyTorch and part of the OpenMMLab project. MMDetection supports a variety of object detection models and allows the deployment of the Swin Transformer model without requiring additional installation packages. Specifically, we used version 2.3.0 of MMDetection, version 2.1.0 of PyTorch, and Python version 3.10.

In this experiment, we used the official Swin Transformer model without any modifications. The server’s CPU was an Intel Xeon (R) Platinum 8362, and the GPU was an RTX 3090 with 24GB of memory. The key training parameters were as follows: the AdamW optimizer with a weight decay of 0.05, a learning rate of 0.0001, and a total of 100 epochs. Learning rate scheduling followed a step policy, with a linear warm-up over 500 steps and a warm-up ratio of 0.001.

For the dataset, we split it into two subsets: set A and set B, in a random 8:2 ratio. Set A served as the training set, while set B was used for validation within the CNN. Additionally, Dataset 5 was employed as an external test set to evaluate the model’s performance. To assess the impact of different MRI sequences on glioma classification, all images were categorized into five groups. For each training, validation, and testing phase, one category was selected for the CNN input.

To address the issue of data imbalance between glioma subtypes, data augmentation techniques were applied to the underrepresented categories. These techniques included rotation, translation, flipping, and scaling, which helped generate additional training samples.

### Statistical analysis

Demographic data and tumor characteristics were calculated statistically for the training, validation and test sets using IMB SPSS 24.0, with *p* < 0.05 indicating a statistically significant difference. Measures (non-normally distributed) were described by median and interquartile spacing; counts were described by frequency and percentage. Multiple group comparisons were made using the chi-square test or Kruskal-Wallis test, and post-hoc two-by-two comparisons were made using the Bonferroni method. The receiver operating characteristic curve (ROC), area under the curve (AUC) of the ROC, Precision, Recall and F1 Score were calculated and generated in the jupyter notebook tool using the python language to evaluate the merit of the model. It is worth noting that while ROC analysis is typically used for binary classification, it can also be applied to evaluate multi-class classification tasks. In this study, we employed a “one *vs.* Rest” strategy, where each class is compared against all other classes, and ROC curves are computed and plotted. This approach treats each class as the positive class, with all other classes combined as the negative class, and the process is repeated for each class. However, in multi-class problems, accuracy may not directly reflect model performance, as it treats all misclassified samples equally, which may not be an accurate representation of real-world scenarios. In contrast, ROC curves and AUC provide a more detailed and nuanced assessment of model performance.

## Results

### Patient characteristics

A total of 811 patients were included in this study, of which 747 patients were from the public data set and 64 patients were from our hospital. There was a significant difference in the age of patients with different types of glioma (*p* < 0.001), with the IDHmut-intact type being younger 40.79 ± 12.88, median age 38.0 (31.0, 50.0); followed by IDHmut-codel 45.08 ± 12.72, median age 47.0 (37.0, 57.0). IDHwt was older 59.98 ± 12.91, median age 60.5 (52.0, 69.0). There was no statistically significant difference in gender between types (*p* = 0.274) (see Table 1).

**Table 1:** General characteristics of participants (*N* = 811).

	Total data *N* = 811	Data 1 *N* = 86	Data 2 *N* = 88	Data 3 *N* = 382	Data 4 *N* = 191	Data 5 *N* = 64
Age, year	53.26 ± 15.44	46.84 ± 14.56	58.32 ± 14.30	55.76 ± 15.59	48.71 ± 14.84	53.59 ± 13.07
Gender						
Men	476 (58.7)	41 (47.7)	56 (63.6)	222 (58.1)	118 (61.8)	39 (60.9)
Female	335 (41.3)	45 (52.3)	32 (36.4)	160 (41.9)	73 (38.2)	25 (39.1)
Grade						
II	196 (24.2)	38 (44.2)	0 (0.0)	44 (11.5)	95 (49.7)	19 (29.7)
III	86 (10.6)	30 (34.9)	0 (0.0)	29 (7.6)	19 (10.0)	8 (12.5)
IV	529 (65.2)	18 (20.9)	88 (100.0)	309 (80.9)	77 (40.3)	37 (57.8)
Type						
IDHwt	490 (60.4)	18 (20.9)	85 (96.6)	286 (74.9)	76 (39.8)	25 (39.1)
IDHmut-intact	105 (32.7)	24 (35.3)	0 (0.0)	14 (14.6)	56 (48.7)	11 (28.2)
IDHmut-codel	216 (67.3)	44 (64.7)	3 (100.0)	82 (85.4)	59 (51.3)	28 (71.8)

IDHwt, IDH-wildtype; IDHmut-intact, IDH-mutant/1p19q-noncodeleted; IDHmut-code, IDH-mutant/1p19q-codeleted. Results shown are median (quartile) or n (%).

### Classification performance

**Figure 2:**
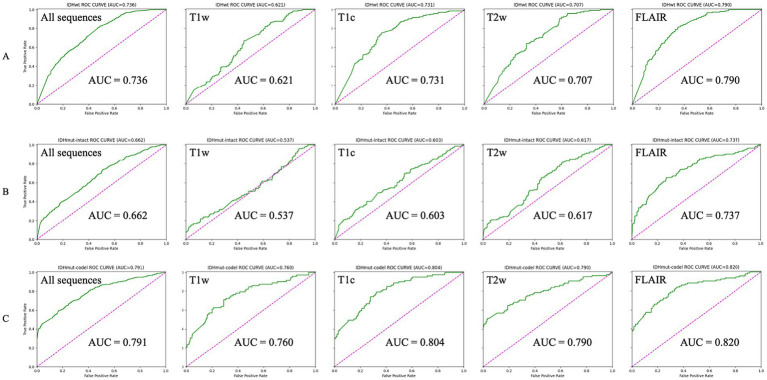
Classification performance of magnetic resonance imaging with different sequences in convolutional neural networks (Test set). **(A–C)** Represent IDHwt (IDH-wildtype), IDHmut-intact (IDH-mutant/1p19q-noncodeleted), and IDHmut-code: IDH-mutant/1p19q-codeleted, respectively. T1w, T1-weighted; T1c, T1-weighted gadolinium contrast-enhanced; T2w, t2-weighted; FLAIR, T2-weighted fluid-attenuated inversion recovery; ALL sequences, T1w + T1c + T2w + FLAIR.

The AUCs for the test sets of IDHwt, IDHmut-intact and IDHmut-codel in the model with all sequences included were 0.736, 0.662 and 0.791, respectively. In the model with only T1w sequences included the test sets AUCs were 0.621, 0.537 and 0.760, respectively. In the model with only T1c sequences included the test sets AUCs were 0.731, 0.603 and 0.804, respectively; in the test set of the model containing only T2w sequences AUCs were 0.707, 0.617 and 0.790, respectively. In the test set of the model containing only FLAIR sequences AUCs were 0.790, 0.737 and 0.820, respectively. The ROCs corresponding to the above AUCs are shown in Figure 2. The ROC and AUC of IDHwt, IDHmut-intact and IDHmut-codel for the training and validation sets of different sequences are shown in the Supplementary Figures 1, 2. The Precision, Recall and F1 Score of IDHwt, IDHmut-intact and IDHmut-codel for the test sets of different sequences are shown in Table 2, the Precision, Recall and F1 Score of IDHwt, IDHmut-intact and IDHmut-codel for the train set and validation set of different sequences are shown in Supplementary Tables 1, 2.

**Table 2:** Classification performance of convolutional neural networks (Test set).

Sequence	Type	Precision	Recall	F1 Score	Average precision
All	IDHwt	49.483	80.369	61.253	57.750
	IDHmut-intact	57.447	40.785	47.703	58.872
	IAHmut-code	82.632	42.432	56.071	66.055
T1w	IDHwt	45.536	73.381	56.198	47.742
	IDHmut-intact	46.154	31.788	37.647	51.527
	IAHmut-code	64.706	30.556	41.509	54.755
T1c	IDHwt	51.786	80.000	62.873	57.307
	IDHmut-intact	51.724	38.961	44.444	52.659
	IAHmut-code	77.500	38.272	51.240	62.424
T2w	IDHwt	44.017	82.400	57.382	50.625
	IDHmut-intact	46.341	25.333	32.759	55.203
	IAHmut-code	46.591	46.591	60.741	70.344
FLAIR	IDHwt	52.107	88.889	65.700	61.732
	IDHmut-intact	70.909	44.828	54.930	69.472
	IAHmut-code	82.000	43.617	56.944	69.414

T1w, T1-weighted; T1c, T1-weighted gadolinium contrast-enhanced; T2w, T2-weighted; FLAIR, T2-weighted fluid-attenuated inversion recovery; ALL, T1w + T1c + T2w + FLAIR. IDHmut-intact, IDH-mutant/1p19q-noncodeleted; IDHwt, IDH-wildtype; IDHmut-code, IDH-mutant/1p19q-codeleted.

### Interpretation of the CNN prediction

**Figure 3:**
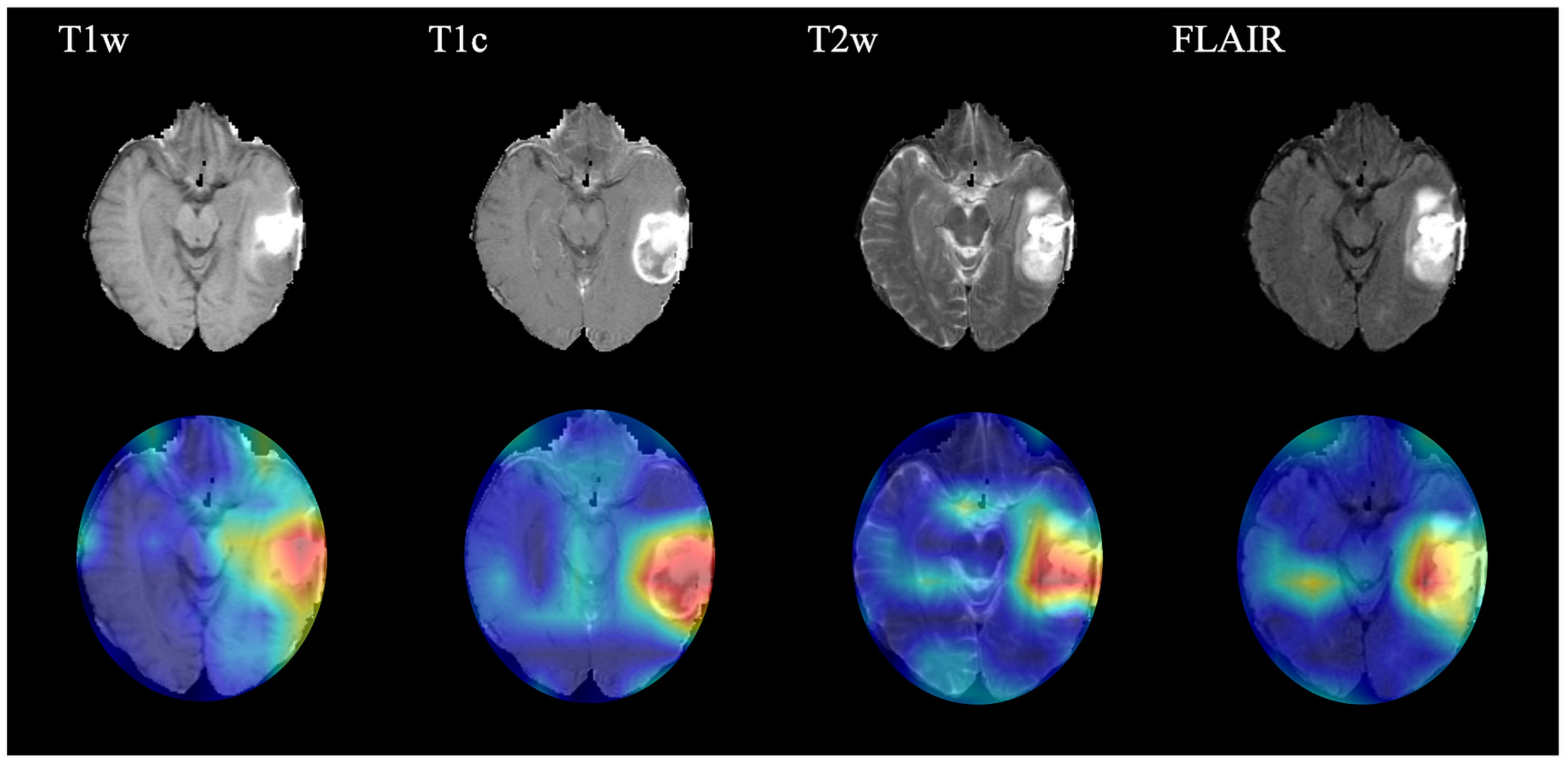
Class activation maps (CAMs) corresponding to four magnetic resonance imaging sequences of a random patient. The CAMs highlighting regions that contribute more to the prediction of glioma type. T1w, T1-weighted; T1c, T1-weighted gadolinium contrast-enhanced; T2w, t2-weighted; FLAIR, T2-weighted fluid-attenuated inversion recovery.

In order to present the key regions identified by CNN for classification of gliomas, four sequences (T1w, T1c, T2w and FLAIR) of a target patient were randomly selected to draw the class activation maps (CAMs) in this study. The CAMs of the corresponding sequences are shown in Figure 3. The results show that the CNN focuses on identifying some regions in the process of classifying gliomas. These images illustrate that the CNN identifies regions that can classify gliomas.

## Discussion

In this paper, we present a novel model capable of effectively predicting the type of glioma prior to surgery. The experiment involved 811 patients, utilizing four public datasets for training and validation of the CNN, while an independent test set was comprised of patients from our hospital. The results demonstrate that the model can accurately classify glioma types across patients from different regions and MRIs obtained from various MRI scanners. Notably, the entire process—from initiating MRI processing for a single patient to generating the final result—takes approximately 10 min. This timeframe includes downloading images from the PACS to the workstation, reviewing the images, preprocessing, manually delineating the ROI, performing image segmentation, and applying the model. The model thus combines the benefits of both time efficiency and high accuracy.

The results of this study support that IDHmut-codel gliomas were significantly the youngest (median 38.0 years), IDHmut-intact (median 47.0 years) was the next youngest, while patients with IDHwt gliomas were generally older (median 60.5 years). Compared to previous studies of glioblastoma, especially before 2021, where there were differences in age characteristics, patients with the IDHwt glioma subtype in this study were younger (22). The main reason for this is that the WHO updated the classification criteria for gliomas as early as 2021 (3) and some IDHwt low-grade gliomas were included in glioblastoma. At the same time the WHO has clarified the importance of IDH and 1p/19q status for the classification of gliomas, and it is on the basis of the classification of glioma IDH and 1p/19q status that the present study was conducted, and it is clear that the demographic results of the present study are more in line with the present day.

Regarding the selection of MRI sequences for CNN training, testing and validation in this experiment, most previous studies have utilized only one of the sequences or a mixture of several sequences, and were unable to do so to explore the impact of different MRI sequences on deep learning results (23, 24). We performed five classifications of four common MRI sequences, T1w, T2w, FLAIR and T1c, as well as a mixture of sequences including the four sequences. We found that the model with input FLAIR sequences had the best classification, with AUCs of 0.790, 0.737 and 0.820 for IDHwt, IDHmut-intact and IDHmut-codel, respectively, and the model with input T1w sequences had the worst classification, with AUCs of 0.621, 0.537 and 0.760, respectively. Considering that different combinations of imaging modalities and the number of images used for model training may further impact classification accuracy, we combined FLAIR images with the other three imaging modalities and tested the mixed dataset. However, the resulting classification performance was still inferior to that achieved with the FLAIR-only dataset. Therefore, we did not proceed with testing other possible combinations of imaging modalities. This is similar to the findings of Jones et al. (25), who showed that models trained with more images did not always perform better. Meanwhile Germann et al. (26). Showed that MRI with different magnetic field strengths did not have a significant effect on the model results. Therefore, after excluding the effect of the number of images and different magnetic field strengths on the classification results, we believe that the FLAIR sequence can be used as the first choice for deep learning for classification tasks.

In this study, the images used for CNN training, validation, and external testing were all manually delineated and cropped ROIs. A few researchers have employed deep learning techniques to address glioma classification tasks using images that are automatically segmented by CNNs (24, 27). The reason we opted for manual delineation rather than developing a multi-task CNN to automatically identify and segment tumors prior to classification is twofold. First, predicting tumor classification remains a significant clinical challenge, while identifying the location and boundaries of tumors is not currently a major obstacle. Second, through the image preprocessing steps in this study, we found that an experienced clinician can quickly and accurately delineate tumor regions, and manual segmentation is a time-efficient process. This approach helps mitigate potential inaccuracies that could arise from discrepancies between automatically segmented images and actual tumor regions. Furthermore, manually segmented images were used for CNN training, testing, and validation to minimize interference from non-tumor elements in the images, which enhances the overall accuracy of the experiment. Of course, with the development and mature application of deep learning in clinical settings, automatic segmentation, with its reproducibility and automation, will be a promising solution.

Regarding the application of deep learning to glioma classification, Choi et al. (28) developed a model to effectively predict the mutational status of IDH in gliomas, but the model only performed binary classification, however, further understanding that IDH mutant gliomas do not undergo 1p/19q co-deletion could provide valuable information for the clinical management of gliomas. Yan et al. (29) used deep learning to effectively predict the 1p/19q co-deletion status of low-grade gliomas, but their study only targeted patients with low-grade gliomas and again only performed binary classification and could not capture or patient IDH status. A comprehensive understanding of patient IDH and 1p/19q co-deletion status would be more useful to clinicians in making treatment decisions.

In fact, a more precise classification of gliomas should include the grade of the tumor after the tumor type is defined, and some scholars have classified tumors according to their grade (30, 31). At the beginning of the experiment, we aimed to incorporate the same criteria for classification of the tumor grade. However, in 2021, the WHO introduced updated classification criteria for gliomas, and IDH wild-type astrocytomas with EGFR amplification, chromosome 7 acquisition with chromosome 10 deletion and TERT promoter mutations are also included in glioblastoma if they have any of the molecular phenotypes. For the current data, we were unable to classify patients by grade according to the 2021 WHO classification criteria, and classification according to the 2016 classification criteria no longer fits the current diagnostic criteria; therefore, we finally abandoned a more recent classification of gliomas by grade.

Despite the results achieved in this study, there are undeniable limitations of this study, firstly, IDH wild type patients accounted for the majority of patients in this study, secondly, four MRI sequences were included in this study. Future research could further explore the impact of other MRI sequences, such as the DWI sequence, which was not examined in this study, on the classification of glioma subtypes. This would represent an interesting avenue for further investigation. Regarding the ROI delineation, the ROIs in this study encompassed the tumor, necrosis and calcifications within the tumor, as well as peritumoral edema. Whether excluding peritumoral edema or using alternative delineation methods could improve classification accuracy remains an area for further investigation.

## Data Availability

The datasets presented in this article are not readily available due to patient privacy. Requests to access the datasets should be directed to Yong Liu, M18856091377@163.COM.

## References

[1] Lapointe S Perry A Butowski NA. Primary brain tumours in adults. Lancet. (2018) 392:432–46. doi: 10.1016/S0140-6736(18)30990-5, PMID: 30060998

[2] Louis DN Perry A Reifenberger G von Deimling A Figarella-Branger D Cavenee WK. The 2016 World Health Organization classification of tumors of the central nervous system: a summary. Acta Neuropathol. (2016) 131:803–20. doi: 10.1007/s00401-016-1545-1, PMID: 27157931

[3] Perez A Huse JT. The evolving classification of diffuse gliomas: World Health Organization updates for 2021. Curr Neurol Neurosci Rep. (2021) 21:67. doi: 10.1007/s11910-021-01153-8, PMID: 34817712

[4] Franceschi E Tosoni A de D Lamberti G Danieli D Pizzolitto S. Postsurgical approaches in low-grade oligodendroglioma: is chemotherapy alone still an option? Oncologist. (2019) 24:664–70. doi: 10.1634/theoncologist.2018-0549, PMID: 30777895 PMC6516106

[5] Aquilanti E Wen PY. Current therapeutic options for glioblastoma and future perspectives. Expert Opin Pharmacother. (2022) 23:1629–40. doi: 10.1080/14656566.2022.2125302, PMID: 36100970

[6] Rossi M Gay L Ambrogi F Conti Nibali M Sciortino T Puglisi G. Association of supratotal resection with progression-free survival, malignant transformation, and overall survival in lower-grade gliomas. Neuro Oncol. (2021) 23:812–26. doi: 10.1093/neuonc/noaa225, PMID: 33049063 PMC8099476

[7] Hervey-Jumper SL Zhang Y Phillips JJ Morshed RA Young JS McCoy L. Interactive effects of molecular, therapeutic, and patient factors on outcome of diffuse low-grade glioma. J Clin Oncol. (2023) 41:2029–2042. doi: 10.1200/JCO.21.02929 36599113 PMC10082290

[8] Motomura K Ohka F Aoki K Saito R. Supratotal resection of gliomas with awake brain mapping: maximal tumor resection preserving motor, language, and neurocognitive functions. Front Neurol. (2022) 13:874826. doi: 10.3389/fneur.2022.874826, PMID: 35645972 PMC9133877

[9] Motomura K Chalise L Ohka F Aoki K Tanahashi K Hirano M. Impact of the extent of resection on the survival of patients with grade II and III gliomas using awake brain mapping. J Neuro-Oncol. (2021) 153:361–72. doi: 10.1007/s11060-021-03776-w, PMID: 34009509

[10] Ostrom QT Shoaf ML Cioffi G Waite K Kruchko C Wen PY. National-level overall survival patterns for molecularly-defined diffuse glioma types in the United States. Neuro Oncol. (2022) 25:799–807. doi: 10.1093/neuonc/noac198, PMID: 35994777 PMC10076944

[11] Yuan F Wang Y Ma C. Current WHO guidelines and the critical role of genetic parameters in the classification of glioma: opportunities for immunotherapy. Curr Treat Options in Oncol. (2022) 23:188–98. doi: 10.1007/s11864-021-00930-4, PMID: 35182297

[12] Gritsch S Batchelor TT Gonzalez Castro LN. Diagnostic, therapeutic, and prognostic implications of the 2021 World Health Organization classification of tumors of the central nervous system. Cancer. (2022) 128:47–58. doi: 10.1002/cncr.33918, PMID: 34633681

[13] Patel SH Poisson LM Brat DJ Zhou Y Cooper L Snuderl M. T2-FLAIR mismatch, an imaging biomarker for IDH and 1p/19q status in lower-grade gliomas: a TCGA/TCIA project. Clin Cancer Res. (2017) 23:6078–85. doi: 10.1158/1078-0432.CCR-17-0560, PMID: 28751449

[14] Li M Ren X Chen X Wang J Shen S Jiang H. Combining hyperintense FLAIR rim and radiological features in identifying IDH mutant 1p/19q non-codeleted lower-grade glioma. Eur Radiol. (2022) 32:3869–79. doi: 10.1007/s00330-021-08500-w, PMID: 35079884

[15] Zhao Q Adeli E Pohl KM. Training confounder-free deep learning models for medical applications. Nat Commun. (2020) 11:6010. doi: 10.1038/s41467-020-19784-9, PMID: 33243992 PMC7691500

[16] Chen S Xu Y Ye M Li Y Sun Y Liang J. Predicting MGMT promoter methylation in diffuse gliomas using deep learning with radiomics. J Clin Med. (2022) 11:3445. doi: 10.3390/jcm11123445, PMID: 35743511 PMC9224690

[17] Pedano N Flanders AE Scarpace L Mikkelsen T Eschbacher JM Hermes B. The cancer genome atlas low grade glioma collection (TCGA-LGG) (Version 3) [Data set]. Cancer Imaging Arch. (2016) doi: 10.7937/K9/TCIA.2016.L4LTD3TK

[18] Bakas S. Segmentation labels for the pre-operative scans of the TCGA-GBM collection [Data set]. Cancer Imaging Arch. (2017). doi: 10.7937/K9/TCIA.2017.KLXWJJ1Q

[19] Clark K Vendt B Smith K Freymann J Kirby J Koppel P. The Cancer imaging archive (TCIA): maintaining and operating a public information repository. J Digit Imaging. (2013) 26:1045–57. doi: 10.1007/s10278-013-9622-7, PMID: 23884657 PMC3824915

[20] Van Der Voort SR Incekara F Wijnenga MMJ Kapsas G Gahrmann R Schouten JW. The Erasmus glioma database (EGD): structural MRI scans, WHO 2016 subtypes, and segmentations of 774 patients with glioma. Data Brief. (2021) 37:107191. doi: 10.1016/j.dib.2021.107191, PMID: 34159239 PMC8203723

[21] Menze BH Jakab A Bauer S Kalpathy-Cramer J Farahani K Kirby J. The multimodal brain tumor image segmentation benchmark (BRATS). IEEE Trans Med Imaging. (2015) 34:1993–2024. doi: 10.1109/TMI.2014.2377694, PMID: 25494501 PMC4833122

[22] Tan AC Ashley DM López GY Malinzak M Friedman HS Khasraw M. Management of glioblastoma: state of the art and future directions. CA Cancer J Clin. (2020) 70:299–312. doi: 10.3322/caac.21613, PMID: 32478924

[23] Hallinan J Zhu L Yang K Makmur A Algazwi DAR Thian YL. Deep learning model for automated detection and classification of Central Canal, lateral recess, and neural Foraminal stenosis at lumbar spine MRI. Radiology. (2021) 300:130–8. doi: 10.1148/radiol.2021204289, PMID: 33973835

[24] Eweje FR Bao B Wu J Dalal D Liao WH He Y. Deep learning for classification of bone lesions on routine MRI. EBioMedicine. (2021) 68:103402. doi: 10.1016/j.ebiom.2021.103402, PMID: 34098339 PMC8190437

[25] Jones AD Graff JP Darrow M Borowsky A Olson KA Gandour-Edwards R. Impact of pre-analytical variables on deep learning accuracy in histopathology. Histopathology. (2019) 75:39–53. doi: 10.1111/his.13844, PMID: 30801768 PMC6591093

[26] Germann C Marbach G Civardi F Fucentese SF Fritz J Sutter R. Deep convolutional neural network-based diagnosis of anterior cruciate ligament tears: performance comparison of homogenous versus heterogeneous knee MRI cohorts with different pulse sequence protocols and 1.5-T and 3-T magnetic field strengths. Investig Radiol. (2020) 55:499–506. doi: 10.1097/RLI.0000000000000664, PMID: 32168039 PMC7343178

[27] Cluceru J Interian Y Phillips JJ Molinaro AM Luks TL Alcaide-Leon P. Improving the noninvasive classification of glioma genetic subtype with deep learning and diffusion-weighted imaging. Neuro-Oncology. (2022) 24:639–52. doi: 10.1093/neuonc/noab238, PMID: 34653254 PMC8972294

[28] Choi YS Bae S Chang JH Kang SG Kim SH Kim J. Fully automated hybrid approach to predict the IDH mutation status of gliomas via deep learning and radiomics. Neuro-Oncology. (2021) 23:304–13. doi: 10.1093/neuonc/noaa177, PMID: 32706862 PMC7906063

[29] Yan J Zhang S Sun Q Wang W Duan W Wang L. Predicting 1p/19q co-deletion status from magnetic resonance imaging using deep learning in adult-type diffuse lower-grade gliomas: a discovery and validation study. Lab Investig. (2022) 102:154–9. doi: 10.1038/s41374-021-00692-5, PMID: 34782727

[30] Xue Z Xin B Wang D Wang X. Radiomics-enhanced multi-task neural network for non-invasive glioma subtyping and segmentation; proceedings of the Radiomics and Radiogenomics in Neuro-oncology: First International Workshop, RNO-AI 2019. Held in conjunction with MICCAI 2019, Shenzhen, China. (2019).

[31] Zhuge Y Ning H Mathen P Cheng JY Krauze AV Camphausen K. Automated glioma grading on conventional MRI images using deep convolutional neural networks. Med Phys. (2020) 47:3044–53. doi: 10.1002/mp.14168, PMID: 32277478 PMC8494136

