# Supplementary material for: Magnetic resonance imaging-based deep learning for predicting subtypes of glioma
Source: Front Neurol. 2025 Jan 29;16:1518815. doi: 10.3389/fneur.2025.1518815 (PMC11813795; doi:10.3389/fneur.2025.1518815)

**Magnetic Resonance Scanner Parameters**

Magnetic resonance imaging (MRI) from our hospital was scanned by two different 3.0T MRI scanners (GE Signal 3.0T and Discovery750W 3.0T) with the following scan parameters:

**T1w**, Tepetition time (TR), 200 ms; Echo time (TE), 75 ms; Number of slices, 18; Section thickness, 6.0 mm; Slices gap, 1.0 mm。

**T1c**, TR, 2500ms, TE, 24 ms; Number of slices, 18; Section thickness, 6.0 mm; Slices gap, 1.0 mm.

**T2w**, TR, 100 ms, TE, 75 ms; Number of slices, 18; Section thickness, 6.0 mm; Slices gap, 1.0 mm.

**FLAIR**, TR8000ms, TE, 121 ms; Number of slices, 18; Section thickness, 6.0 mm; Slices gap, 1.0 mm.

MRIs from the TCGA-LGG dataset were obtained from 3.0T MRI scanners from three manufacturers (General Electric, Philips and SIEMENS) and 1.5T MRI scanners from three manufacturers (General Electric, Philips and SIEMENS) with the following scan parameters:

**T1w**, TR, 366-3257 ms; TE, 2-20 ms; Inversion time (IE), 0-1100 ms; Number of slices, 20-309; Flip angle (FA), 9°-150°.

**T1c**, TR, 5-3230 ms; TE, 2.1-20 ms; IE, 0-1238 ms; Number of slices, 16-451; FA, 9°-90°.

**T2w**, TR, 883-6000 ms; TE, 16-120 ms; IE, 0 ms; Number of slices, 9-411; FA, 90°-180°.

**FLAIR**, TR, 6000-11000 ms; TE, 94-155 ms; IE, 2000-2850 ms; Number of slices, 20-351; FA, 90°-180°.

The MRIs in the TCGA-GBM dataset are from 3.0T MRI scanners from three manufacturers (General Electric, Philips and SIEMENS) and 1.5T MRI scanners from three manufacturers (General Electric, Philips and SIEMENS) with the following scan parameters:

**T1w**, TR, 5-3380 ms; TE, 2-19 ms; IE, 0-1238 ms ; Number of slices, 20-359; FA, 8°-150°.

**T1c**, TR, 5-3286 ms; TE, 2-20 ms; IE, 0-1238 ms; Number of slices,16-339; FA, 8°-90°.

**T2w**, TR, 2020-6050 ms; TE, 13-355 ms; IE, 0 ms; Number of slices, N20-144; FA, 90°-180°.

**FLAIR**, TR, 1000-11000 ms; TE, 74-155 ms; IE,1900-2850 ms; Number of slices, 20-178; FA, 90°-180°.

Specific MRI scanner parameters are not listed in this paper as they are not available for other datasets.

**Supplementary Table 1 Classification performance of convolutional neural networks (Train set).**

| **Sequence** | **Type** | **Precision** | **Recall** | **F1 Score** | **Average Precision** |
| --- | --- | --- | --- | --- | --- |
| All | IDHwt | 92.621 | 99.106 | 95.753 | 99.430 |
|  | IDHmut-intact | 98.281 | 90.352 | 94.150 | 98.523 |
|  | IAHmut-code | 97.213 | 88.446 | 92.622 | 96.755 |
| T1w | IDHwt | 90.610 | 98.934 | 94.589 | 99.300 |
|  | IDHmut-intact | 97.566 | 90.076 | 93.671 | 98.633 |
|  | IAHmut-code | 97.604 | 87.408 | 92.225 | 96.964 |
| T1c | IDHwt | 94.870 | 99.503 | 97.131 | 99.619 |
|  | IDHmut-intact | 98.353 | 91.236 | 94.661 | 98.472 |
|  | IAHmut-code | 98.159 | 89.463 | 93.612 | 96.902 |
| T2w | IDHwt | 92.920 | 99.167 | 95.942 | 99.473 |
|  | IDHmut-intact | 98.655 | 90.411 | 94.353 | 98.623 |
|  | IAHmut-code | 95.681 | 87.702 | 91.518 | 96.337 |
| FLAIR | IDHwt | 91.710 | 98.686 | 95.070 | 99.306 |
|  | IDHmut-intact | 97.746 | 90.293 | 93.872 | 98.549 |
|  | IAHmut-code | 97.545 | 89.447 | 93.320 | 97.309 |

*Note:* T1w: T1-weighted; T1c: T1-weighted gadolinium contrast-enhanced; T2w: T2-weighted; FLAIR: T2-weighted fluid-attenuated inversion recovery; ALL: T1w + T1c + T2w + FLAIR. IDHmut-intact: IDH-mutant / 1p19q-noncodeleted; IDHwt: IDH-wildtype; IDHmut-code:IDH-mutant / 1p19q-codeleted.

**Supplementary Table 2 Classification performance of convolutional neural networks (Validation set)**

| **Sequence** | **Type** | **Precision** | **Recall** | **F1 Score** | **Average Precision** |
| --- | --- | --- | --- | --- | --- |
| All | IDHwt | 91.727 | 98.677 | 95.075 | 99.262 |
|  | IDHmut-intact | 97.192 | 88.993 | 92.912 | 98.088 |
|  | IAHmut-code | 96.269 | 86.294 | 91.009 | 95.992 |
| T1w | IDHwt | 90.613 | 98.195 | 94.252 | 99.120 |
|  | IDHmut-intact | 96.530 | 91.005 | 93.686 | 98.504 |
|  | IAHmut-code | 97.172 | 85.520 | 90.975 | 96.783 |
| T1c | IDHwt | 93.961 | 99.169 | 96.494 | 99.514 |
|  | IDHmut-intact | 98.002 | 89.485 | 93.550 | 98.020 |
|  | IAHmut-code | 94.148 | 85.648 | 89.697 | 95.010 |
| T2w | IDHwt | 92.582 | 99.031 | 95.698 | 99.460 |
|  | IDHmut-intact | 97.694 | 88.268 | 92.742 | 98.213 |
|  | IAHmut-code | 94.602 | 88.462 | 91.429 | 96.645 |
| FLAIR | IDHwt | 91.389 | 98.515 | 94.818 | 99.207 |
|  | IDHmut-intact | 96.985 | 90.407 | 93.581 | 98.354 |
|  | IAHmut-code | 97.269 | 87.030 | 91.865 | 96.760 |

*Note:* T1w: T1-weighted; T1c: T1-weighted gadolinium contrast-enhanced; T2w: T2-weighted; FLAIR: T2-weighted fluid-attenuated inversion recovery; ALL: T1w + T1c + T2w + FLAIR. IDHmut-intact: IDH-mutant / 1p19q-noncodeleted; IDHwt: IDH-wildtype; IDHmut-code:IDH-mutant / 1p19q-codeleted.

**Supplementary Fig 1. Classification performance of magnetic resonance imaging with different sequences in convolutional neural networks (Train set).** A, B and C represent IDHwt (IDH-wildtype), IDHmut-intact (IDH-mutant / 1p19q-noncodeleted), and IDHmut-code (IDH-mutant / 1p19q-codeleted), respectively. T1w: T1-weighted; T1c: T1-weighted gadolinium contrast-enhanced; T2w: t2-weighted; FLAIR: T2-weighted fluid-attenuated inversion recovery; ALL sequences: T1w + T1c + T2w + FLAIR.


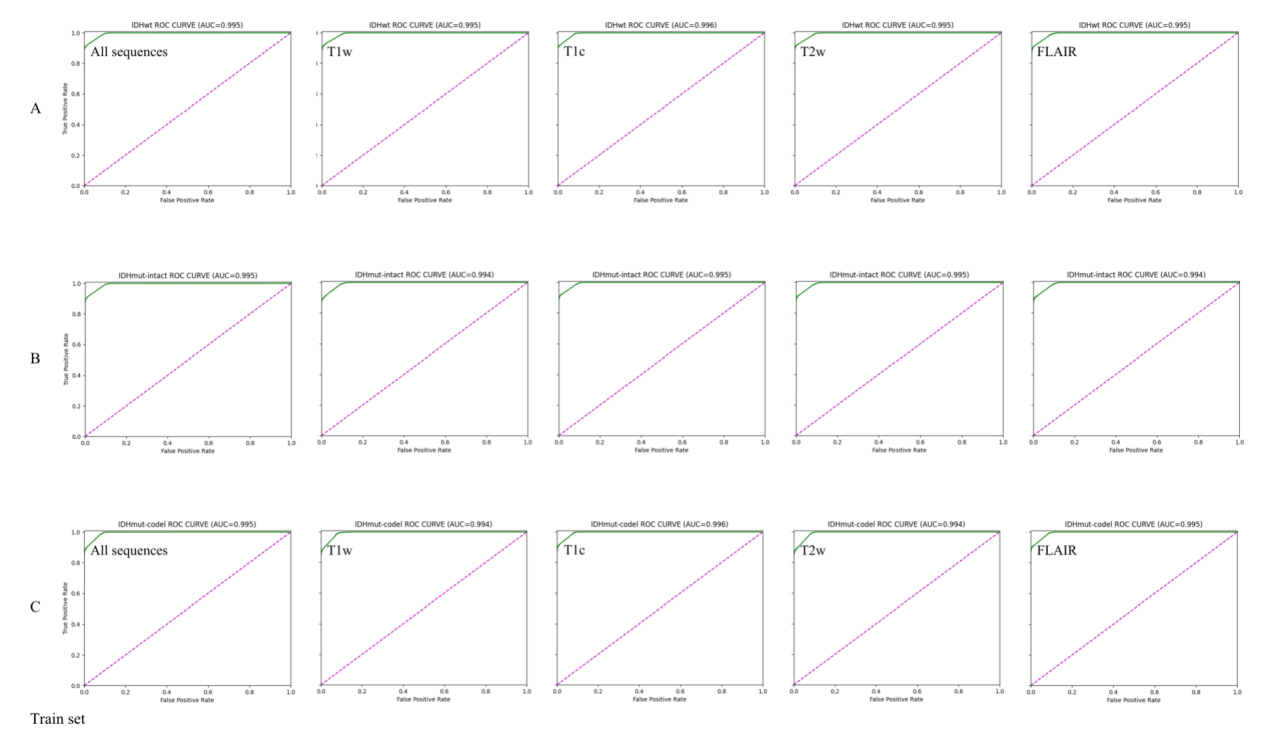


**Supplementary Fig 2.** **Classification performance of magnetic resonance imaging with different sequences in convolutional neural networks (Validation set).** A, B and C represent IDHwt (IDH-wildtype), IDHmut-intact (IDH-mutant / 1p19q-noncodeleted), and IDHmut-code (IDH-mutant / 1p19q-codeleted), respectively. T1w: T1-weighted; T1c: T1-weighted gadolinium contrast-enhanced; T2w: t2-weighted; FLAIR: T2-weighted fluid-attenuated inversion recovery; ALL sequences: T1w + T1c + T2w + FLAIR.


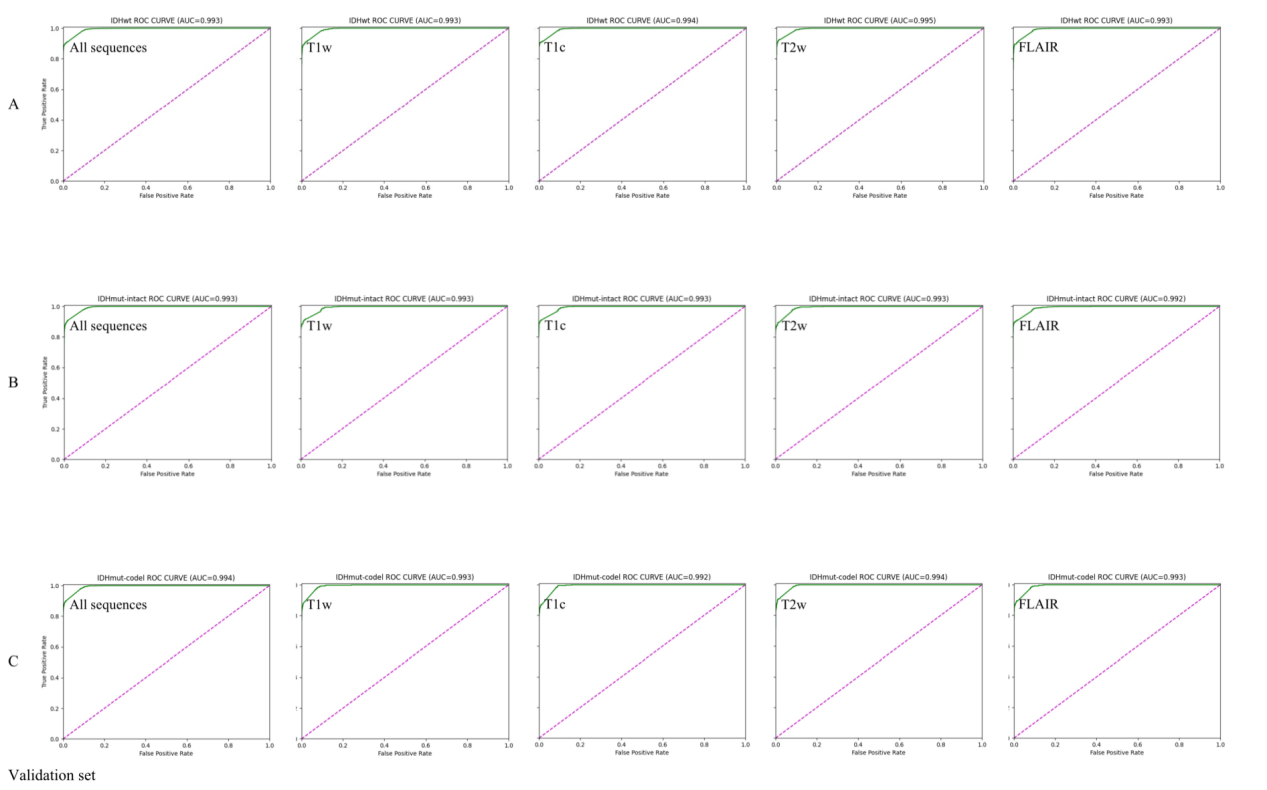

Supplement: Supplementary file 1 [file Table_1.docx]
